# Medication reviews in hospitalized patients: a qualitative study on perceptions of primary and secondary care providers on interprofessional collaboration

**DOI:** 10.1186/s12913-020-05744-y

**Published:** 2020-09-29

**Authors:** Bregje Walraven, Godelieve Ponjee, Wieke Heideman, Fatma Karapinar Çarkit

**Affiliations:** 1 Department of Clinical Pharmacy, OLVG, Jan Tooropstraat 164, 1061 AE Amsterdam, the Netherlands; 2 Present address: Department of Psychiatry, GGZ in Geest, Haarlem, the Netherlands; 3 Department of Clinical Pharmacy, Amsterdam UMC location AMC, Amsterdam, the Netherlands; 4 Department of Research and Epidemiology, OLVG, Amsterdam, Netherlands

**Keywords:** Medication review, Medication therapy management, Polypharmacy, Interprofessional relations, Continuity of care

## Abstract

**Background:**

In-hospital medication reviews are regularly performed. However, discontinuity in care could occur because secondary care providers lack insight into the outpatient history. Furthermore, for the implementation or follow-up of some medication review-based interventions, the help of primary care providers is essential. This requires interprofessional collaboration between secondary and primary care. Therefore, the aim of this qualitative study was to gain insight into the perceptions of primary and secondary care providers on interprofessional collaboration on medication reviews in hospitalised patients.

**Methods:**

Ten face-to-face semi-structured interviews and three focus group discussions were conducted with 20 healthcare providers from three hospitals and community health services. The interviews were aimed at exploring general practitioners’, community pharmacists’, geriatricians’, and hospital pharmacists’ experiences, attitudes, and views of interprofessional collaboration. Focus groups consisted of representatives of all professional groups. Through group discussion, interprofessional collaboration was explored by addressing three main questions: 1) What are the benefits of in-hospital medication reviews? 2) What are the barriers to in-hospital medication reviews from an interprofessional collaboration perspective? 3) Given the barriers mentioned, how should this interprofessional collaboration between primary and secondary care be designed? Data were analysed using a thematic-content approach.

**Results:**

The need for in-hospital medication reviews was underlined due to their many benefits, such as reducing potentially preventable re-admissions. Barriers regarding interprofessional collaboration between primary and secondary care can be subdivided into three main themes: 1) defining in-hospital medication reviews (e.g., lack of clear goals), 2) execution of medication reviews (e.g., hospital setting is dynamic), and 3) follow-up after discharge (e.g., unclear instructions). Care providers suggested solutions for each of the barriers mentioned, for example, by using supportive staff in order to overcome the gap between primary and secondary care providers and making clear agreements on proper means of communication.

**Conclusion:**

Primary and secondary care providers recognise the importance of in-hospital medication reviews and the need for interprofessional collaboration. To create satisfying interprofessional collaboration, conditions should be met on defining in-hospital medication reviews across settings and involving both primary and secondary care providers in implementing medication reviews and organising their follow-up.

## Background

According to the World Health Organisation (WHO), in 2050 more than one in five individuals will be aged over 60 years [1]. This older population is more likely to suffer from polypharmacy [2, 3] (i.e., the use of five or more medications [4]. Unindicated polypharmacy can result in increased healthcare costs, adverse drug events, medication non-adherence, decrease in functional status, and even death [5–7]. Therefore, much effort is put into reducing unindicated polypharmacy in the older population, such as by performing medication reviews. A medication review is defined as a structured evaluation of a patient’s medicines with the aim of optimising medicine use and improving health outcomes [8]. Initially, in Australia, the United Kingdom, and the Netherlands, primary care providers (primary-CP) — i.e., general practitioners (GPs) and community pharmacists (CPs) — were called upon to introduce protocols for conducting medication reviews [3, 9, 10]. In addition, the need for secondary care providers (secondary-CPs) to perform in-hospital reviews arose also, as 20% of readmissions within 30 days of hospital discharge are related to medication, [11] and performing medication reviews in primary care hardly affects these hospital readmissions. Also, hospitalised patients likely represent a frailer patient group compared with patients in primary care [12]. Indeed, a recent systematic review showed that medication related harm after discharge was common [13] and stressed the need for in-hospital medication reviews. Many studies have evaluated in-hospital medication reviews [12, 14]. A recent Cochrane meta-analysis based on 10 trials evaluating the effectiveness of in-hospital medication reviews shows the great variety in performing such a review [12], such as who performed this review, how to inform primary-CPs of the changes suggested, or how to organise follow-up. This review did not show a reduction in mortality or hospital readmission, which could be interpreted as a barrier toward implementing in-patient medication reviews. However, the authors were reluctant to rule out a beneficial effect because of the described heterogeneity of the included studies. A recent study by Nielsen et al. [15] showed the importance of collaboration across settings in in-hospital medication reviews. This study showed that a reduction in hospital readmissions was present only in a subgroup of patients receiving an extended medication review as opposed to a basic intervention. The extended medication review also included contact with a primary-CP by detailed discharge papers and a follow up phone call. Therefore, the authors stress the need for more interprofessional collaboration as primary-CPs follow-up on interventions performed or suggested by secondary-CPs. However, no previous research has focused on the perspectives of these care providers on this interprofessional collaboration.

Previous qualitative studies on interprofessional collaboration in performing medication reviews have focused solely on primary-CPs and show the importance of having a clear system [16] and mutual recognition of roles and expectations [17–20]. Now that secondary-CPs are also called upon by guidelines to perform medication reviews, new systems, roles and expectations arise, like how to organise inquiring background information regarding the patient, what follow-up actions are needed post-discharge, and responsibilities of the healthcare providers.

Therefore, the aim of this study was to gain insight into the perceptions of primary- and secondary- CPs on interprofessional collaboration on medication reviews in hospitalised patients.

## Method

### Study design

This multi-step qualitative study combined face-to-face semi-structured interviews with focus groups in order to reach methodological triangulation [21]. A qualitative design was chosen to investigate perceptions of the professionals, allowing to explore attitudes, context and different perspectives. The face-to-face semi-structured interviews contributed to individual opinions and experiences, whereas the focus groups explored areas of consensus and discrepancy using group dynamics [22]. The COREQ checklist [23] was used as a guideline for qualitative studies. Approval was obtained from the local committee of the hospital (OLVG hospital (ID WO:18.179) and written informed consent was obtained from all participants.

### Participants

In total, 30 health care providers were recruited from three hospitals (OLVG, Amsterdam UMC and BovenIJ) and community health services located in the city of Amsterdam, the Netherlands. Secondary-CPs were represented by geriatricians, and pharmacists working in hospital (hospital pharmacists or outpatient clinic pharmacists) based on the interventions described in the Cochrane meta-analysis on medication reviews in hospitalised patients [12]. Primary-CPs were represented by GPs an CPs, because they are currently responsible for the medication reviews in primary care [3, 9, 10]. A variation-covering representativeness was created by purposive sampling, taking into account a variation in gender and years of experience in the field of work. All participants were selected from within the network of the researchers combined with snowball sampling. Participants were approached by email, telephone or both. There were no personal relations between the interviewer/facilitator and participants.

### Data collection and analysis

Collection, analysis, and discussion of data alternated between each step (see Table 1).

**Table 1:** Study flow using the multi-step approach and methodological triangulation

Steps		Result	Discussion in research team
**Preparation interviews**	Construction of a topic list based on literature research and team discussions.	Development of the topic list for the interviews	The research team agreed on the topic list.
**Exploratory face-to-face interviews with primary-CP**	Explore perceptions of general practitioners and community pharmacists. Themes discussed were prior experiences, attitudes and views on interprofessional collaboration between primary- and secondary-CP when performing in-hospital medication reviews.	Primary-CP have little experience in performing medication reviews in secondary care. However, due to previous experiences in collaborating with secondary-CP, they can imagine both advantages and barriers of performing medication reviews in secondary care.	Secondary-CP should be included in order to complete the actors involved in the interprofessional collaboration.
**Exploratory face-to-face interviews with secondary-CP**	Explore perceptions of (internist)-geriatricians and pharmacists working in hospital. Themes discussed were identical to the ones discussed in step 2.	Secondary-CP have some experience in performing medication reviews in secondary care. They also identify advantages and barriers. These are partially identical to the ones primary-CP mentioned, but differ at some points.	Focus groups should be held in order to explore areas of consensus and discrepancy between both settings using group dynamics.
**Meeting first- and second coder**	Discuss differences in coding of all interviews between first- and second coder, until consensus was reached.	Development of a coding structure	The research team agreed on the developed coding structure.
**Preparation focus groups**	Combine the literature search with themes mentioned in the exploratory interviews, resulting in a topic list	Development of the topic list for the focus groups	The research team agreed on the topic list.
**Focus group 1**	Explore perceptions of primary- and secondary-CP using three main questions: 1. What are the benefits of in-hospital medication reviews? 2. What are the barriers concerning interprofessional collaboration between primary and secondary care? and 3. Given the barriers mentioned, how should this interprofessional collaboration be designed?	New codes and themes arose during coding. First coder (BW) updated the coding structure.	The next focus group should focus more on the barriers concerning interprofessional collaboration between primary and secondary care
**Focus group 2**	Explore perceptions further on the three themes (see step 6). Emphasis lies on barriers concerning interprofessional collaboration between primary and secondary care.	New codes and themes arose on barriers concerning interprofessional collaboration. First coder updated the coding structure.	The next focus group should focus more on solutions on how interprofessional collaboration should be designed.
**Focus group 3**	Explore perceptions further on the three themes (see step 6). Emphasis lies on how interprofessional collaboration should be designed.	No new codes and themes arose.	First coder suggested that saturation was reached, independently
**Meeting first- and second coder**	Second coder (GP) performed second coding independently. Discuss differences in coding between first- and second coder until consensus was reached.	Development of a final coding structure	Second coder agreed on data saturation. The research team agreed on the final coding structure. This was then applied to all transcripts.

Interviews were performed by two medical students, who were trained and supervised by experienced research team members. Interviews with primary-CPs were performed in June- and July 2017 by student 1, whereas interviews with secondary CPs were performed by student 2 in January 2019 due to change in capacity. No major changes in policy occurred on performing Dutch in-hospital medication reviews between 2017 and 2019 which therefore limits the effect of this time gap. The discussion guide used in interviews and focus groups was developed for this study and is provided as Additional File 1.

Topics discussed during interviews were prior experiences, attitudes, and views on interprofessional collaboration between both settings when performing in-hospital medication reviews.

Three focus groups with representatives of primary- and secondary care were led by BW and FC in February- and March 2019. To facilitate discussion between primary- and secondary-CPs three questions were addressed: 1) *What are the benefits of in-hospital medication reviews*? 2) *What are the barriers concerning interprofessional collaboration between the professionals?* 3) *Given the barriers mentioned, how should this interprofessional collaboration be designed?*

All interviews and focus groups were audio taped and transcribed verbatim. Field notes were made in order to describe contextual details and non-verbal expressions during focus groups. To ensure correct interpretation, member checking for all focus groups was performed by sending a summary to all participants. Data were collected until saturation was reached, determined when no new themes were found.

Coding and categorising of transcripts were executed by two coders independently performing a thematic-content approach using MAXQDA 2007.

Differences in coding were regularly discussed and themes were reviewed thoroughly until consensus was reached in the research team. This resulted in a final coding structure (Additional file 2). The final coding structure was then applied to all transcripts.

## Results

A total of 66 care providers were invited to the study; 25 (38%) refused and 6 (9%) did not respond. The main reasons for refusal were lack of interest (*n* = 7) or no time (*n* = 18). In total, 35 care providers agreed to participate in the study, and 5 care providers withdrew at the last minute due to personal reasons, resulting in a total of 30 participants. The demographic data of the participants are provided in Table 2.

**Table 2:** Demographic data of the respondents. Including last minute cancellations

Groups	General practitioner (GP)		Community pharmacist (CP)		Geriatrician *		Pharmacist working in hospital (HP) **	
**Participated**	9		7		7		7	
**Approached**	23		13		21		9	
	*Interviews*	*Focus groups*	*Interviews*	*Focus groups*	*Interviews*	*Focus groups*	*Interviews*	*Focus groups*
**Participants**	3	6	3	4	2	5	2	5
**Gender**								
*Male*	1	3	2	2		1		2
*Female*	2	3	1	2	2	4	2	3
**Years of experience**								
*0–5*		1	1		1	2	1	4
*5–10*	1	1				1		
*10–20*	2	3	1	3	1	1		
*> 20*		1	1	1		1	1	1

*consisting of 5 geriatricians and 2 internist-geriatricians. **: consisting of 4 hospital pharmacists and 3 outpatient clinic pharmacists working in hospital

On average, the duration of the interviews was 27 min (range: 18–47 min) and the focus groups 109 min (range: 100–119).

### Benefits of performing medication reviews in secondary care

In interviews, several positive experiences in collaboration between primary- and secondary-CPs for in-hospital medication reviews were mentioned. These experiences were explored further in focus groups revealing several benefits of performing medication reviews in secondary care (see Table 3).

**Table 3:** Benefits of performing medication reviews in secondary care, mentioned by care providers in both interviews and focus groups

**Benefits of performing medication reviews in secondary care**	
Performing medication reviews is part of providing good care (e.g., trying to reduce readmissions) *‘Well, I think the most important argument is that it is part of proper conduct, preventing continuing damage ... Of course, it would be great if that does not happen again.’ (Geriatrician, 5–10 years of experience [yrs], focus group [FG] 3)*	
Performing medication reviews could be complementary to primary care *‘But of course, there are people who slip through the cracks, for example due to new facts that are not yet known.’ (Geriatrician, 5–10 yrs, FG 3)*	
A hospital setting has advantages compared to the primary care setting - Access to hospital expertise and diagnostic tools - Access to multiple specialists or consulting parties - Possibility of monitoring patients when implementing medication changes - More time available during hospital admission as opposed to the consulting setting of primary care - Using the hospital admission as motivation for implementing changes *‘That is the advantage of secondary care. That you are, of course, present 24 h a day to see how that patient is doing.’ (Pharmacist working in hospital [HP], < 5 yrs, FG 3)*	
Education for young physicians *‘I really do notice that when you’re an assistant in training, this is very useful for the rest of your career. You are collecting knowledge*.’ (*GP, 10–20 yrs, FG 1)*	
Creating a complete patient file after discharge *‘I think that if you do a medication assessment before discharging a patient, you create ample stability for after the patient has been discharged and establish clarity for the GP, the public pharmacy, and the specialist and paint a complete picture.’ (CP, 10–20 years of experience, FG 1)*	

### Barriers and solutions for interprofessional collaboration

**Fig. 1:**
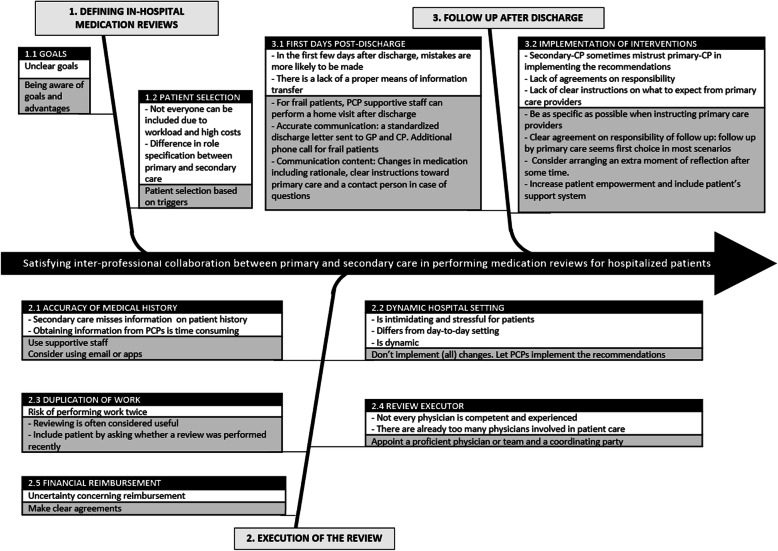
Barriers and solutions for implementing interprofessional collaboration on in-hospital medication reviews. This can be subdivided into three main themes: 1) defining medication reviews, 2) execution of the review, and 3) after discharge and solutions on how to deal with the barriers mentioned

Barriers to implementing interprofessional collaboration on in-hospital medication reviews were discussed during interviews and focus groups. Also, solutions were brought up in the focus groups. These barriers and solutions can be divided into three themes — *1) defining in-hospital medication reviews*, *2) execution of the medication review*, and *3) follow-up after discharge* — as shown in Fig. 1.

#### Defining in-hospital medication reviews

This theme concerns thoughts on the definition of in-hospital medication reviews and includes the subthemes of *defining* a *goal* and *patient selection*.

##### Goals

Caregivers described medication reviews as a systematic assessment of the pharmacotherapy resulting in an optimisation of an individual’s medication. However, for some primary-CPs, the specific goal for in-hospital medication reviews was unclear.

*‘So, I would really like to invest extra time in this, but in advance, I would like to know what the problem is that we are dealing with [...] What are we going to solve together?’ (GP, 10–20 yrs, interview).*

Interestingly, during the focus groups, care providers were aware of many benefits and could describe goals in performing in-hospital medication reviews, as showed in Table 3.

##### Patient selection

Not everyone can be included in an in-hospital medication review due to workload and high costs in secondary care. Both primary- and secondary-CPs point out that the hospital should not take over a primary care function.

*‘Well, I can imagine that some GPs will be rather offended by that. They will be like “How is that any of your business?”’ (CP, > 20 yrs, interview).*

In order to overcome this barrier, caregivers suggested that secondary- CPs should only focus on the more complex cases or include patients based on risk factors for drug-related (re) admissions, high-risk medications, and risk factors for frailty.

#### Execution of the medication review

This theme concerns the actual execution of an in-hospital medication review. Five subthemes emerged: *accuracy of medical history*, *dynamic hospital setting*, *duplication of work*, *review executor*, and *financial reimbursement*.

##### Accuracy of medical history

Care providers explained that for in-hospital medication reviews, collecting information from primary care is time-consuming, but essential (e.g., current medication, medical history including previously tried therapies, social and personal factors, and therapy adherence).

*‘So that...that type of information is the type I find very time-consuming and annoying. But that is essential. Otherwise, you cannot assess medication.’ (Geriatrician, < 5 yrs, FG 2).*

During two focus groups, it was suggested to use trained supportive staff in order to obtain this information. Also, primary-CPs suggested using email or apps to improve efficiency.

*‘Yes, you could send an email in that case [...] Perhaps you should not say anything besides: “we’re going to discuss polypharmacy, are there any specifics?” And then you will hear: adherence is hard, informal care is difficult. Then we will bring up things that are not necessarily bound to medication or are actually bound to it, for example “don’t touch those benzos.”’ (GP, 10–20 yrs, FG 1).*

##### Dynamic hospital setting

Care providers identified that the hospital setting differs from the home setting: patients might be intimidated and stressed by the hospital, and the situation of hospitalised patients is dynamic (e.g., a decrease in blood pressure due to a gastro-intestinal infection).

In order to overcome this barrier, GPs suggested that not all recommendations should be implemented immediately (e.g., decrease dose of an antihypertensive drug). Instead, recommendations should be directed towards primary care and implemented in the home setting (e.g., when the blood pressure is stable). Secondary-CPs worried that GPs would feel that the hospitals throw work over the fence, but this was not recognised by GPs in the focus groups.

*‘But when it very clearly says “advice for the GP”. That makes me so happy. Because then I know “Oh yes, this is what I must do and I can turn it into a pop-up and write it down in my planner.”’ (GP, < 5 yrs, FG 3).*

All care providers agree that changes should be made immediately in the case of acute threats (e.g., kidney failure) or when the momentum is right (e.g., motivation of patient to taper off benzodiazepines).

##### Duplication

All professions mentioned the risk of potentially performing work twice. Nevertheless, not all participants experienced this as a barrier. In the case of a medication-related admission, performing a medication review was considered essential:

*‘If you are a doctor and you are responsible for your patient, I think you are obligated to seriously consider their medication. And I do not see the harm in that, because the situation can change ever so slightly two months later, and it might be necessary to repeat the assessment.’ (GP, 5–10 years of experience, FG 2).*

When asked for possible solutions, particularly primary-CPs noted to make the selection clear and act pragmatically. Primary-CPs could exclude patients for reviews in primary care if patients were recently hospitalised with many medication changes. Another solution was to ask patients whether they had received a review recently and needed another one.

##### Review executor

Both primary- and secondary-CPs fear that less competent physicians (e.g., due to lack of training, experience, and/or motivation) take part in in-hospital medication reviews. A second barrier mentioned was including yet another physician in taking care of one patient.

According to care providers, appointing a competent physician or team who could assist and coordinate when necessary could be useful in order to address this barrier. Members of this team could be hospital pharmacists, internists, geriatricians, or pharmacologists interested in polypharmacy.

##### Financial reimbursement

Concerning financial reimbursement, care providers fear two things. First, in interviews, CPs fear the financial consequences of the hospital partly taking over their jobs.

*‘Most pharmacies here in Amsterdam are independent owners [...] And as such, you are not terribly thrilled when some kind of club suddenly drops in and goes “Oh, we will just take care of that.”’ (CP, > 20 yrs, interview).*

Second, the possible scenario is feared in which only one party will be reimbursed in the case of both primary- and secondary-CPs performing a review around the same period of time. In order to address this barrier, care providers stated that clear agreements need to be made concerning reimbursement.

#### Follow-up after discharge

This theme includes the follow-up phase after discharge. Two subthemes emerged: *first days post-discharge* and *implementation of interventions*.

##### First days post-discharge

Many care providers stated that the transition from hospital to home is a high-risk period for drug-related harm because of medication changes and poor transfer of information to primary care.

*‘Two blood pressure medications were stopped because she experienced a fall, and this was not fully communicated to the pharmacy, so she still got those pills [...] Or perhaps the patient has not fully understood the changes, and so she kept taking all the medications she always took the next day’ (GP, < 5 years of experience, FG 3).*

Primary-CPs suggested using supporting staff, such as community nurses, for a home visit for frail patients. Even though some secondary-CPs were reluctant to request this, both GPs and CPs stated that they are willing to provide this service if necessary.

Within the information transfer, both the means and the content offer barriers. Because crucial information gets lost or forgotten within the large amount of paperwork, three possible means of communication were suggested. First of all, secondary-CPs should use a defined format, alerting the GP and CP that a review has been performed.

*‘When you see how many letters we receive on a daily basis, a lot slips through the cracks .... And when it arrives separately, it has far more chance of catching your attention.’ (GP, 5–10 years of experience, FG 2).*

The second means of communication is a follow-up phone call to the patient’s primary-CP. Care providers admit that this is a time-consuming intervention, but direct contact is considered essential, especially for complex patients. A third way is using digital patient files. However, privacy issues could arise, and the ideal system has not been developed yet.

Regarding the content of the information transfer, primary-CPs mention three things being mandatory: reason for medication changes, clear instructions on what is expected, and a contact person for questions.

*‘It is very unpleasant for someone who is talking to the doorman and does not know who in the house they need to speak to. And five phone calls later, people find themselves losing it just a little, or getting angry.’ (Geriatrician, > 20 yrs, FG 1).*

##### Implementation of interventions

Some interventions need to be implemented post-discharge. Many (internist-) geriatricians describe the experience of sending proposed changes to primary-CPs but find out that changes were not implemented. This leads to mistrust toward primary-CPs.

*‘I also have a large amount of distrust towards how much of the advice originating from secondary care is actually followed up by primary care. And they can all be great reasons, but they can also be bad reasons.” (Geriatrician, > 20 years of experience, FG 1).*

In order to address this, primary-CPs point out the need to be specific when instructing primary care.

*‘You can really help GPs when it says what it is expected of GPs. When I find that in letters, it is a pleasant read. Because then I am like “That is what I’m going to do.”’ (GP, 10–20 yrs, FG 2).*

Also, clear agreements are needed on who is responsible for following up. Three scenarios were discussed. The first scenario is following up on all suggested changes by primary care. This is considered favourable because primary-CPs feel responsible for patients in a home setting.

*‘Because afterwards, they are discharged and they are our responsibility again.’ (GP, 10–20 years of experience, interview).*

Follow-up could be conducted by secondary-CPs for frail or complicated patients or when patients were already followed up by a hospital physician. A third option is a fusion between primary and secondary care: secondary-CPs could move into the primary care scene and vice versa. Nevertheless, this is thought to be very time-consuming.

*‘In a perfect world, I think I would maybe have someone from the second line sit down with the GP for some kind of pharmacotherapeutic discussion … To preserve continuity or advice.*’ *(Geriatrician, 10–20 yrs, interview).*

Moreover, the follow-up phase should also contain a moment of reflection (e.g., are all recommendations implemented and are therapy goals reached?). There was no mention of a distinct favourable option on whether this should be carried out by primary- or secondary-CPs.

*‘Or that we implement some check-up after the fact to check whether our advice was followed, so we can still step in if needed. This could also be a follow-up consultation by phone or by a nurse specialist, or a pharmacist or, uh, a video consultation if [laughs] you want to go for something more modern.’ (Geriatrician, 10–20 yrs, interview).*

Also, increasing empowerment of patients and their caregivers is important. Methods mentioned were instructing patients and carers when to reach out and toward whom and explaining the changes made using teach-back methods, frequent repetitions, and enabling patients to review their own patient file.

*‘The patient should be properly informed on why something is happening. If the patient is properly informed, they can also take on the role of a … guardian. When something goes wrong in primary care.’ (HP, > 20 yrs, interview).*

## Discussion

This qualitative study aimed to gain insight into the perceptions of primary- and secondary- CPs on interprofessional collaboration on medication reviews in hospitalised patients.

Both primary- and secondary-CPs see the added value of in-hospital medication reviews, as they mentioned similar benefits like access to expertise and diagnostic tools and the possibility of monitoring patients when implementing medication changes. Barriers regarding interprofessional collaboration between primary- and secondary care were, for example, the dynamic hospital situation or lack of insight into the outpatient medical history and home situation. Clear agreements on patient selection, responsibilities of primary and secondary care, and communication need to be addressed first to create a successful interprofessional collaboration.

Prior literature on performing medication reviews in primary care underlines the need for clear specification of roles and responsibilities for GPs and CPs [17–20, 24]. This study shows that this is also important for in-hospital medication reviews, as secondary care should not take over a primary care role, but select a population suited for an in-hospital medication review, based on triggers. We also found that primary-CPs are willing to implement the recommendations suggested in secondary care. This is important because the decreasing length of hospital stays [25, 26] results in less time available for implementation and evaluation of medication changes. Also, a hospital setting may be a suboptimal setting due to stress or illnesses resulting in physical changes such as increased blood pressure [27] or a decrease in the patient’s cognitive function [28]. Therefore, implementation of interventions following a medication review could take place in the more stable home setting.

Furthermore, previous research on medication reviews in primary care showed that effective and open communication is fundamental [17, 18, 24]. This study underlines that adequate interprofessional communication in in-hospital medication reviews is also important as two moments of communication are considered essential: 1) prior to a review, to gather information about the outpatient history and to discuss potential interventions, and 2) after the review, to communicate the hospital-based recommendations and make agreements on the implementation, monitoring, and follow-up. In the studies included in the Cochrane meta-analysis on the effectiveness of in-hospital medication reviews, these communication moments are not fully implemented yet [12]. This might be a reason why previous studies have failed to show clear effects of in-hospital medication reviews on patient outcomes.

Previous research on performing medication reviews in secondary care implies that an improvement of clinical outcomes can occur only if primary care is included [12, 15]. To our knowledge, this is the first qualitative study seeking the perspectives of primary- and secondary-CPs on interprofessional collaboration. Moreover, this study is an addition to the previous literature that focused on primary care only [17–20, 24]. Another strength of this study is the triangulation in data collection, as data were collected from both interviews and focus groups. Also, the multi-step character of this study resulted in more detailed results due to slight adjustments that were made in between steps.

There are also a few limitations to this study. First, this study included only care providers in the proximity of Amsterdam, limiting the generalisability. However, in order to minimise this limitation, purposive sampling was used [22]. Also, in other countries such as Belgium, Denmark, the USA and Sweden [12], similar situations occur in which in-hospital reviews are performed, suggesting that these findings could also be implemented elsewhere.

Second, only care providers who agreed to participate were included, resulting in the absence of attitudes of those who declined to participate. In order to address this limitation, interviews were used in addition to focus groups to enable participants to also address their concerns, as participants of interviews tend to be more honest instead of giving the desirable answers in comparison with focus groups [22]. Lastly, patients and carers were not included in this study, as we focused on the interprofessional collaboration between settings. Future studies should also focus on how patients experience in-hospital medication reviews and what barriers and solutions they see. Also, further research could focus more implementation of the suggested solutions and test its feasibility.

## Conclusion

Primary- and secondary-CPs recognise the importance of in-hospital medication reviews and the need for interprofessional collaboration. To create a satisfying interprofessional collaboration, conditions should be met on defining in-hospital medication reviews across settings and involving both primary- and secondary-CPs in implementing medication reviews and organising their follow-up. These findings have implications for both policy makers and researchers. Further research is needed to determine how best to embed these interventions efficiently into existing processes.

## Supplementary information

**Additional file 1.** Interview guide

**Additional file 2 Code system**

## Data Availability

The datasets used and/or analysed during the current study are available from the corresponding author on reasonable request.

## Abbreviations

Primary-CP: Primary care provider
Secondary-CP: Secondary care provider
GP: General practitioner
CP: Community pharmacist
HP: Pharmacist working in hospital
FG: Focus group
Yrs: Years of experience
