# Supplementary material for: Medication reviews in hospitalized patients: a qualitative study on perceptions of primary and secondary care providers on interprofessional collaboration
Source: BMC Health Serv Res. 2020 Sep 29;20:902. doi: 10.1186/s12913-020-05744-y (PMC7526422; doi:10.1186/s12913-020-05744-y)
Supplement: Supplementary file 1 — Additional file 1. Interview guide [file 12913_2020_5744_MOESM1_ESM.docx]

**Additional file 1: interview guide**

Interview guide: interviews

- Have you ever experienced a situation in which a medication review in hospital was performed?
- Do you think there are benefits of performing medication reviews in hospital? Can you tell me why?
- Which factors can make the implementation of performing medication reviews in hospital more of less of a success?
- Would you like to be involved in the process of performing medication reviews in hospital? In which way?
- What do you think is important in the communication between primary and secondary care providers in performing medication reviews in hospital?

Interview guide: focus group 1

1. What are the benefits of performing medication reviews in hospital? Put these on green post its
2. What are the barriers or disadvantages of performing medication reviews in hospital? Put these on red post its
3. *The red and green post its are put on the wall*. Put a sticker on what you think is the most important benefit and the most important barrier.
4. Group discussion: First reactions from the audience. Do people agree with everything? Do people disagree? Are there differences or similarities between professional groups?
5. *The focus group facilitator takes the red post its with the barriers and puts them on the wall.* Multiple barriers were mentioned. I would like to brainstorm with you on how we could deal with these barriers and focus on solutions through group discussion and exploratory questions.

Interview guide: focus group 2

In the second focus group, the research team decided that more focus should be on the barriers concerning interprofessional collaboration between primary and secondary care. Therefore, the interview guide changed slightly:

1. *The green post its of the benefits of performing medication reviews in hospital, mentioned in the first focus groups were put on the wall*. First reactions from the audience. Do you miss anything? Do people agree with everything? Do people disagree? Are there differences or similarities between professional groups?
2. What are the barriers or disadvantages of performing medication reviews in hospital? Put these on red post its
3. *The red post its are put on the wall*. Put a sticker on what you think is the most important barrier.
4. Group discussion: First reactions from the audience. Do people agree with everything? Do people disagree? Are there differences or similarities between professional groups?
5. *The focus group facilitator takes the red post its with the barriers and puts them on the wall.* Multiple barriers were mentioned. I would like to brainstorm with you on how we could deal with these barriers and focus on solutions through group discussion and exploratory questions.

Interview guide: focus group 3

In the third focus group, the research team decided that more focus should be on solutions on how interprofessional collaboration should be designed. Therefore, the interview guide changed slightly:

1. *The green and red post its of the benefits and barriers of performing medication reviews in hospital, mentioned in the first two focus groups were put on the wall*. First reactions from the audience. Do you miss anything? Do people agree with everything? Do people disagree? Are there differences or similarities between professional groups?
2. *The focus group facilitator takes the red post its with the barriers and puts them on the wall.* Multiple barriers were mentioned. I would like to brainstorm with you on how we could deal with these barriers and focus on solutions through group discussion and exploratory questions.
