# Supplementary material for: Medication reviews in hospitalized patients: a qualitative study on perceptions of primary and secondary care providers on interprofessional collaboration
Source: BMC Health Serv Res. 2020 Sep 29;20:902. doi: 10.1186/s12913-020-05744-y (PMC7526422; doi:10.1186/s12913-020-05744-y)
Supplement: Supplementary file 2 — Additional file 2 Code system [file 12913_2020_5744_MOESM2_ESM.docx]

**Additional file 2: code system**

Advantages of performing medication reviews in secondary care

Performing medication reviews is part of providing proper care

Performing medication reviews could be complementary to PC

Advantages of hospital setting

Using the hospital admission as motivation for implementing cha

Access to expertise and diagnostic tools

Access to multiple specialist or consulting parties

Possibility of monitoring patients when implementing changes

More time available in a hospital admission

Informative for young physicians

Creating a complete patient file after discharge

Barriers and solutions of performing medication review in SC

Defining reviews

Goals

Patient selection

Time pressure and high costs in secondary care

Difference in role specification between primary- and secondary

Demarcation between primary- and secondary care population

Cause of admission

Number or type of medication

Risk factors for frailty

Execution of the review

Accuracy of medical history

Secondary care misses out on information originating from prima

Current medication

Medical history including previously tried therapies

Social and personal factors

Therapy adherence

Method of inquiring information

Using supportive staff

Proper communication prior to a review is important

Dynamic hospital setting

Hospital setting seems suboptimal

Patients might be intimidated and stressed out

Hospital setting differs from a day-to-day setting

The situation of hospitalized patients is too dynamic

Advisory role of SC in stead of implementing all changes

Duplication of work

Risk of performing work twice

Reduce this risk

Re-doing reviews can be useful

Include patient

Primary- CP exclude patients which had recently been admitted

Financial reimbursement

Reimbursement toward the patient

Reimbursement/competition between care providers

Review executor

Not every physician is competent

Too many physicians involved in care of one patient

Appointing a physician/team for assistance and coordination

Follow up after discharge

First days post discharge

High risk period for drug related harm

Problems due to poor information transfer

Problems due to medication changes

Use supportive staff

Proper information transfer

Means of information transfer

Using a standardized format

Electronic files

Discharge letter

Phone call

Important information gets lost in the transfer

Content of information transfer

Medication changes+rationale, instructions PC, contact person

Implementation of interventions

Failure of follow up by primary care

Mistrust towards primary care

Lack of agreement on responsibility

Lack of clear instructions towards primary care

Be specific when instructing primary care.

Responsibility for follow up

Follow up by a fusion of primary and secondary care

Follow up by secondary care

Follow up by primary care

Including an extra moment of reflection

Empowerment of patient and support system
